# Curcumol Targets the VHL/HIF-1α Axis to Suppress Glycolysis-Driven Progression in Colorectal Cancer

**DOI:** 10.3390/cancers17183000

**Published:** 2025-09-14

**Authors:** Gang Wang, Zengyaran Yue, Gang Yin, Lifeng Zhu, Wen Zhou, Ruiqian Sun, Tingting Bi, Lin Zhao, Yong Bian, Decai Tang

**Affiliations:** 1 School of Chinese Medicine, Nanjing University of Chinese Medicine, Nanjing 210023, China; 2 School of Medicine, Nanjing University of Chinese Medicine, Nanjing 210023, China042121223@njucm.edu.cn (L.Z.); 3 Nanjing Hospital of Chinese Medicine, Nanjing University of Chinese Medicine, Nanjing 210023, China; 4 School of Integrative Medicine, Nanjing University of Chinese Medicine, Nanjing 210023, China; 5 School of Pharmacy, Nanjing University of Chinese Medicine, Nanjing 210023, China; 6 Laboratory Animal Center, Nanjing University of Chinese Medicine, Nanjing 210023, China

**Keywords:** colorectal cancer (CRC), HIF-1α, Curcumol, metabolic reprogramming

## Abstract

**Background**: Hypoxia-induced glycolysis represents a hallmark of colorectal cancer (CRC) progression and contributes significantly to therapeutic resistance. Curcumol, a natural sesquiterpenoid derived from Curcumae Rhizoma, has demonstrated promising anti-tumor properties. However, its impact on metabolic reprogramming under hypoxic conditions remains largely undefined. **Objective**: The objective of this study was to elucidate the potential of Curcumol in inhibiting glycolytic reprogramming and impede CRC progression via regulation of the VHL/HIF-1α signaling pathway. **Methods**: CRC cells and orthotopic mouse models were treated with Curcumol under chemically induced hypoxic conditions. Metabolic alterations were evaluated using Seahorse extracellular flux analysis, Western blot analysis, quantitative real-time PCR (qRT-PCR), immunohistochemistry (IHC) and co-immunoprecipitation (Co-IP). Functional validation of glycolysis and epithelial–mesenchymal transition (EMT) phenotypes was conducted through in vitro and in vivo assays. **Results**: Curcumol inhibited HIF-1α-mediated metabolic reprogramming by upregulating VHL expression, thereby promoting HIF-1α degradation. This effect led to the downregulation of key glycolytic genes (HK2, LDHA, and GLUT1), decreased glycolytic flux, and lactate production, ultimately suppressing CRC cell proliferation and invasion. The anti-tumor efficacy of Curcumol was validated in both in vitro and in vivo models. Moreover, Curcumol effectively reversed the hypoxia-induced epithelial–mesenchymal transition (EMT) phenotype, suggesting that its metabolic regulatory effects may contribute to reduced metastatic potential. **Conclusions**: Curcumol suppresses glycolysis and CRC progression by activating the VHL/HIF-1α signaling axis. These findings underscore the potential of Curcumol as a natural metabolic regulator capable of reversing tumor metabolic reprogramming, offering a promising therapeutic strategy for CRC treatment.

## Simple Summary

Colorectal cancer (CRC) cells often adapt to low-oxygen environments by increasing glycolysis, which promotes tumor growth and metastasis. This study shows that Curcumol, a natural compound from Curcumae Rhizoma, can block this process by activating the VHL/HIF-1α pathway. Curcumol reduces glycolytic activity, suppresses tumor cell invasion, and reverses EMT, both in cells and animal models. These findings suggest that Curcumol may serve as a promising natural agent for CRC treatment.

## 1. Introduction

Colorectal cancer (CRC) is one of the most prevalent malignancies worldwide and remains a leading cause of cancer-related mortality [1,2]. Despite advances in early detection and screening, more than 50% of CRC cases are diagnosed at advanced stages, where treatment options are limited and prognosis remains poor [3]. Although chemotherapy has extended survival rate in certain cases, its overall efficacy remains unsatisfactory due to frequent emergence of therapeutic resistance [4]. Moreover, the recurrence rate reaches approximately 20% within five years after surgery, further contributing to persistently high mortality associated with CRC [5,6,7]. These clinical challenges underscore the urgent need for novel and effective therapeutic strategies, particularly for advanced and metastatic CRC.

One of the hallmarks of tumor progression is metabolic reprogramming, which enables cancer cells to adapt and thrive under hypoxic conditions. CRC cells often exhibit increased oxygen consumption, resulting in localized hypoxia within the tumor microenvironment [8,9]. In response, hypoxia-inducible factor-1 alpha (HIF-1α), a master transcriptional regulator of cellular metabolism, becomes aberrantly stabilized and overexpressed [10]. HIF-1α promotes a metabolic shift from oxidative phosphorylation to glycolysis—known as the Warburg effect—allowing tumor cells to maintain energy production despite oxygen deficiency [11]. Under normoxic conditions, HIF-1α is rapidly hydroxylated and targeted for proteasomal degradation via the Von Hippel–Lindau (VHL) pathway. However, genetic or functional loss of VHL leads to the accumulation of HIF-1α, which subsequently upregulates key glycolytic enzymes such as hexokinase-2 (HK2) and lactate dehydrogenase A (LDHA), promoting glucose uptake and lactate production, ultimately enhancing tumor proliferation and metastatic potential [12,13].

Given the central role of HIF-1α in metabolic reprogramming and tumor progression, it has emerged as a promising therapeutic target in CRC [14,15]. Curcumol, a sesquiterpenoid product extracted from Curcuma species, has demonstrated notable anti-cancer activity in various malignancies, partly by destabilizing HIF-1α under hypoxic conditions [16,17]. Mechanistically, Curcumol has been shown to modulate multiple upstream regulators of HIF-1α stabilization, including STAT3, mTOR, and MAPK signaling pathways [18,19]. By downregulating HIF-1α, Curcumol disrupts critical metabolic processes such as glycolysis and glutaminolysis, both of which are essential for CRC progression [20].

Therefore, our study aimed to investigate whether Curcumol suppresses CRC progression by targeting the VHL/HIF-1α axis and modulating hypoxia-induced metabolic reprogramming. Our findings might offer a novel mechanistic rationale for the therapeutic application of Curcumol in the treatment of advanced or metastatic CRC [14,21].

## 2. Methods

### 2.1. Curcumol Preparation and Administration

The Curcumol (≥98% purity) used in this study was purchased from Shanghai Yuanye Bio-Technology Co., Ltd., Shanghai, China. (Catalog No. B20342). For experimental use, Curcumol was initially dissolved in sterile DMSO to prepare stock solutions. In cell-based assays, the final concentration of DMSO was maintained below 0.1% to avoid solvent-related effects. For animal experiments, Curcumol was dissolved in 0.9% sodium chloride injection solution.

### 2.2. Animal Studies and Treatment

BALB/C mice (6–8 weeks, 20 ± 2 g) were obtained from the Experimental Animal Resource Center of Nanjing University of Chinese Medicine and raised under specific pathogen-free (SPF) conditions. All animal procedures were approved by the Ethics Committee of Nanjing University of Chinese Medicine (Approval No. 202207A047, License No. SYXK (Jiangsu) 2023-0077) and conducted in accordance with NIH guidelines.

To establish the CRC orthotopic model, CT26 cells (Procell, Wuhan, China, CL-0071) or VHL-knockout CT26 cells (constructed via LentiCRISPR v2, Antyhera Biotech, Xiamen, China) were subcutaneously injected into the axillary region of mice (Table 1). For VHL knockout, a lentiviral CRISPR–Cas9 system was employed using the LentiCRISPR v2 vector carrying a VHL-targeting guide RNA. Lentiviral particles were packaged and transduced into CT26 cells following standard procedures, and stable clones were selected using puromycin (2 μg/mL). Successful VHL deletion was confirmed by Western blot analysis and qRT-PCR prior to in vivo implantation. Once tumors reached a size of about 100 mm^3^, they were excised and cut into 1 mm^3^ cubes, then orthotopically implanted onto the cecum wall under isoflurane anesthesia. Curcumol was administered intraperitoneally at 80 mg/kg/day for 14 consecutive days. Mice were randomly divided into four groups: control, VHL-KO, Curcumol, and VHL-KO + Curcumol.

### 2.3. Transcriptomic Analysis and Correlation Assessment of Mouse Tumor Tissues

Total RNA was extracted from fresh mouse tumor tissues using TRIzol reagent. RNA quality was assessed with Agilent 2100 Bioanalyzer (Agilent, Nanjing, China), and samples with RIN > 7.0 were selected for library preparation and paired-end sequencing on the Illumina NovaSeq 6000 platform (Nanjing, China). Sequencing data were filtered with Trimmomatic, aligned to the mouse reference genome (GRCm39) using HISAT2 and quantified as FPKM values via StringTie (V3.0). Pearson correlation analysis was conducted using the cor() function in R Programming Language (Rstudio, R4.3.3) to evaluate the expression relationship between VHL and EMT-related genes. The results were visualized using heatmaps.

### 2.4. CRC Organoids

#### 2.4.1. Organoid Derivation

Biopsy tissues from CRC patients were transported on ice in PBS containing penicillin and streptomycin, washed, and minced into about 2 mm^3^ fragments. Tissues were digested in Gentle Cell Dissociation Reagent (STEMCELL, Shanghai, China, #07174) with gentle oscillation on ice for 30 min, filtered through a 70 μm strainer, and pelleted via centrifugation. The cell pellet was resuspended in cold PBS and mixed with growth factor-free Matrigel (Corning, Nanjing, China, #356231), then seeded into 24-well plates and incubated at 37 °C for 1 h for gel polymerization. Each well was supplemented with 500 μL of IntestiCult™ Organoid Growth Medium (STEMCELL, Shanghai, China, #06010) containing apoptosis inhibitor Y27632 (STEMCELL, #72304). This study was approved by the Ethics Committee of Nanjing Hospital of Chinese Medicine Affiliated to Nanjing University of Chinese Medicine (Approval No. KY2023323), and all participants provided informed consent.

#### 2.4.2. Organoid Viability Assay

Organoids were seeded into 96-well plates, and cell viability was assessed using the CellTiter-Glo ATP Assay Kit (Laitongshengwu, G7572, Beijing, China) following the manufacturer’s protocol. Luminescence intensity was measured with a microplate reader to evaluate the metabolic activity of organoids.

#### 2.4.3. H&E Staining and Immunohistochemistry (IHC)

Organoids were fixed, paraffin-embedded, and sectioned (5 μm) for hematoxylin and eosin (H&E) staining to assess morphology. For IHC, sections underwent antigen retrieval, blocking, and incubation with Ki67 primary antibody (Abcam, Hangzhou, China), followed by HRP-conjugated secondary antibody and DAB chromogenic detection. For immunofluorescence (IF), sections were co-stained with Ki67 and DAPI and imaged using a Zeiss LSM 880 confocal microscope (Nanjing, China).

### 2.5. Hypoxia Simulation in Cell Culture

The CT26 (Procell, Nanjing, China, CL-0071) and MC38 (Procell, CL-0972) cell lines, both derived from murine colorectal cancer, were used for in vitro experiments. To simulate hypoxic conditions, cells in the logarithmic growth phase were treated with CoCl_2_ at a final concentration of 100 μM and incubated for 24 h to induce stable HIF-1α expression. Cells in the control group were cultured in standard culture medium without CoCl_2_. After treatment, the cells were immediately subjected to subsequent experimental procedures.

### 2.6. Hematoxylin and Eosin (H&E) Staining

Tumor tissues were fixed in 4% paraformaldehyde, embedded in paraffin, and sectioned. Hematoxylin and eosin (H&E) staining was performed for morphological assessment. For immunohistochemistry (IHC), sections underwent antigen retrieval, blocking, and incubation with primary antibodies (e.g., anti-HIF-1α) followed by HRP-conjugated secondary antibodies and DAB visualization.

### 2.7. Western Blot Analysis

Proteins were extracted using lysis buffer (Beyotime, STS0003, Chinae, Shanghai, China), quantified, and resolved via SDS-PAGE. After being transferred to PVDF membranes, samples were blocked with 5% BSA and incubated with primary antibodies overnight at 4 °C, including HIF-1α (#36169), HK2 (#2867), PKM2 (#4053) (Cell Signaling Technology, Beijing, China); GLUT1 (21829-1-AP), LDHA (21799-1-AP) (Proteintech, Wuhan, China). This was followed by secondary antibodies and ECL detection the next day.

### 2.8. Seahorse X 96 Metabolic Flux Analysis

Metabolic function was assessed using a Seahorse XFe96 Analyzer (Nanjing, China, Agilent Technologies). CT26 and MC38 cells (1 × 10^4^/well) were seeded into XF96 plates and subjected to extracellular acidification rate (ECAR) and oxygen consumption rate (OCR) measurements. Glucose, oligomycin, FCCP, and rotenone/antimycin A were sequentially injected to evaluate glycolysis and mitochondrial function.

### 2.9. Immunofluorescence Assay

Cells were seeded on coverslips, and tumor sections were deparaffinized. After fixation, permeabilization, and blocking, samples were incubated with primary antibodies overnight, followed by fluorescent secondary antibodies and DAPI counterstaining. Images were acquired using a Leica THUNDER system (Nanjing, China) and analyzed using Leica LAS.

### 2.10. RT-PCR

Total RNA from tumor tissues was extracted using an RNA isolation kit (Vazyme, RC102-01, Nanjing, China) and reverse-transcribed into cDNA. Real-time PCR was conducted using SYBR Green Master Mix and a Roche LightCycler 96 (Nanjing, China). Primers targeting HIF-1α, HK2, PKM2, GLUT1, and LDHA were synthesized by Sangon Biotech (Sangon Biotech Co., Ltd., Shanghai, China). Gene expression levels were calculated using the 2^−ΔΔCt^ method (Table 2).

### 2.11. Transwell Invasion Assay

Cells (2 × 10^5^/well) were seeded in collagen-coated inserts and cultured in serum-free medium. After 24 h, invaded cells were fixed, stained with crystal violet, and counted.

### 2.12. Wound Healing Assay

Confluent cells were scratched with a sterile pipette tip, treated with Curcumol, and imaged at 0 h and 24 h.

### 2.13. Colony Formation Assay

Cells were seeded at a density of 1000 per well and treated with Curcumol. After 10 days, colonies were fixed, stained, and counted.

### 2.14. Metabolic Analysis

The levels of glycolysis-related metabolites, including glucose-6-phosphate (G6P), pyruvate, lactate, phosphoenolpyruvate (PEP), and ATP, were measured using commercial kits (Jiancheng Bioengineering Institute, Nanjing, China; Catalog Nos.: SDZ 500 M015-1-1, A081-1-1, A019-2-1, A131-1-1, A095-1-1) according to the manufacturer’s protocols.

### 2.15. Co-IP

Cells were lysed using IP buffer (Beyotime), and lysates were incubated overnight with VHL or HIF-1α antibodies. Protein A/G magnetic beads (Beyotime) were then added, and the complex were cultured for 2 h at 4 °C. Immunoprecipitates were washed and then analyzed via Western blot as described above.

### 2.16. Statistical Analysis

All data are presented as means ± SD and analyzed using SPSS 16.0. Significant differences were determined by one-way ANOVA and post hoc Tukey’s test. Values of *p* < 0.05 were considered statistically significant.

## 3. Results

### 3.1. Curcumol Inhibits Proliferation and Migration of CRC Cells Under Hypoxia

**Figure 1:**
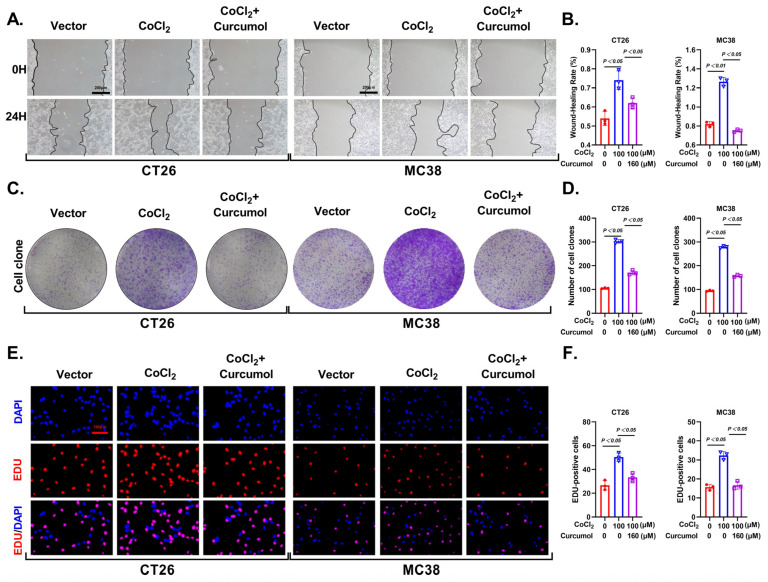
Curcumol inhibited proliferation and migration of CRC cells under hypoxia. Cells were treated with Curcumol at indicated concentrations for 24 h (**A**–**D**) or 10 days (**E**,**F**). (**A**,**B**) Wound healing assay and its quantification. (**C**,**D**) Colony formation assay with quantification. (**E**,**F**) EdU Assay. The results are presented as means ± SD. All experimental military repetitions three times (n = 3). Significance: CoCl_2_ vs. vector, CoCl_2_ vs. CoCl_2_ + Curcumol.

To evaluate the anti-tumor potential of Curcumol under hypoxic conditions, we first established a chemically induced hypoxia model in colorectal cancer (CRC) cells. Specifically, the cells were treated with 100 μM of CoCl_2_ for 24 h to stimulate a hypoxic tumor microenvironment and create a stable low-oxygen condition. The subsequent experiments were all conducted under these hypoxic conditions. Wound healing and colony formation assays showed that hypoxia significantly enhanced CRC cell proliferation and migration, whereas Curcumol treatment markedly suppressed these effects (Figure 1A–D). Furthermore, EdU incorporation assays confirmed the inhibitory effect of Curcumol on cell proliferation (Figure 1E,F). In summary, the above findings suggest that Curcumol effectively inhibits hypoxia-driven tumor growth and motility.

### 3.2. Curcumol Suppresses the Invasion and Metastasis of CRC Cells

**Figure 2:**
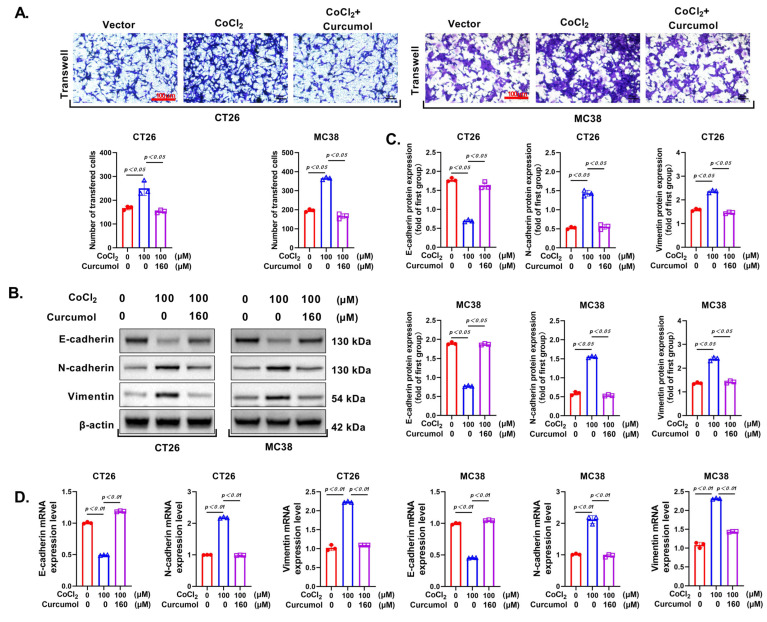
Curcumol inhibited proliferation and migration of CRC cells. Cells were treated with Curcumol at indicated concentrations for 24 h. (**A**) Transwell invasion assay. (**B**,**C**) Western blot analysis of E-cadhernin, N-cadherinin, and Vimentin with quantification. (**D**) Real-time PCR analysis of E-cadhernin, N-cadherinin, and Vimentin with quantification. The results are presented as means ± SD. All experimental military repetitions three times (n = 3). Significance: CoCl_2_ vs. vector, CoCl_2_ vs. CoCl_2_ + Curcumol. The original images of the Western Blotting figures can be found in Supplementary Figure S1.

Since invasiveness is a critical hallmark of cancer progression, we first evaluated whether Curcumol could attenuate the metastatic potential of CRC cells. Transwell invasion assays revealed that the invasive capacity of CT26 and MC38 cells was significantly enhanced under hypoxia-mimicking conditions (CoCl_2_ treatment), whereas Curcumol treatment markedly reduced cell invasion (Figure 2A). Further analysis of Western blot and qRT-PCR demonstrated that CoCl_2_ exposure led to a significant downregulation of E-cadherin and upregulation of N-cadherin and Vimentin, while Curcumol treatment effectively restored E-cadherin expression and suppressed N-cadherin and Vimentin levels (Figure 2B–D), indicating that Curcumol inhibits the epithelial–mesenchymal transition (EMT) process. These findings suggest that Curcumol suppresses the invasive phenotype of CRC cells by reversing EMT.

### 3.3. Curcumol Inhibits Glycolytic Metabolic Pathway

**Figure 3:**
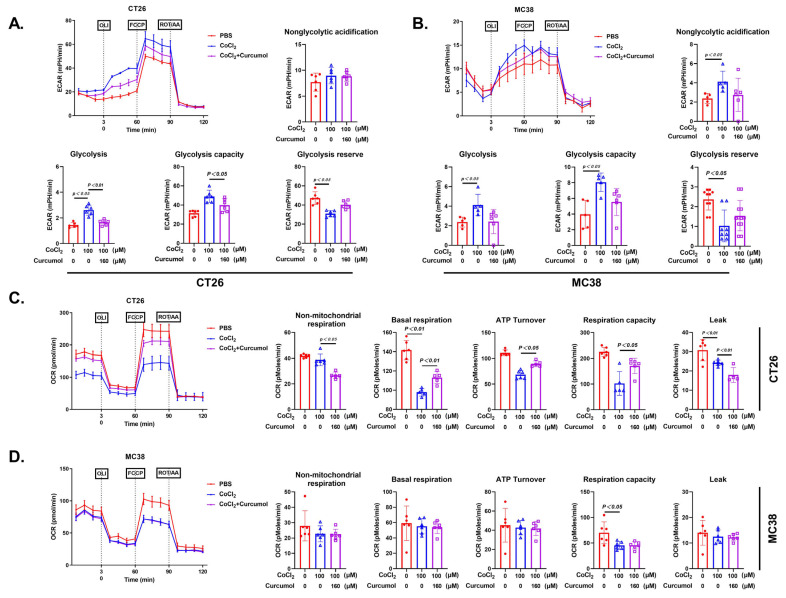
Curcumol inhibited the glycolytic metabolic pathway. Cells were treated with Curcumol at indicated concentrations for 24 h. (**A**) CT26 ECAR with quantification. (**B**) MC38 ECAR with quantification. (**C**) CT26 OCR with quantification. (**D**) MC38 OCR with quantification. The results are presented as means ± SD. All experimental military repetitions three times (n = 3). Significance: CoCl_2_ vs. vector, CoCl_2_ vs. CoCl_2_ + Curcumol.

**Figure 4:**
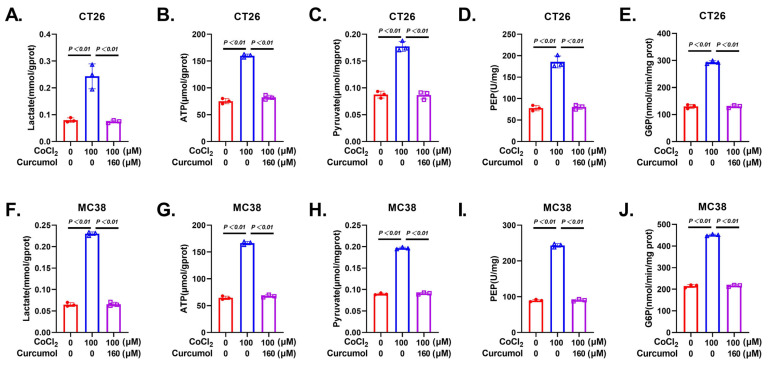
Curcumol inhibited glycolysis products in CRC cells. Cells were treated with Curcumol at indicated concentrations for 24 h. (**A**,**F**) Lactate analysis. (**B**,**G**) ATP analysis. (**C**,**H**) Pyruvate analysis. (**D**,**I**) PEP analysis. (**E**,**J**) G6P analysis. Significance: CoCl_2_ vs. vector, CoCl_2_ vs. CoCl_2_ + Curcumol. The results are presented as means ± SD. All experimental military repetitions three times (n = 3).

Tumor cell invasion and migration heavily depend on metabolic reprogramming, particularly the enhancement of glycolytic activity, which not only provides energy (ATP) but also generates various biosynthetic intermediates [22,23,24]. Based on our previous findings that Curcumol suppresses CRC cell proliferation and migration, we hypothesized that it may affect cellular glycolytic metabolism. Seahorse metabolic analysis revealed that CoCl_2_ treatment significantly increased the extracellular acidification rate (ECAR), while Curcumol treatment markedly reduced ECAR and elevated the oxygen consumption rate (OCR), suggesting a metabolic shift from aerobic glycolysis toward oxidative phosphorylation (Figure 3). Further metabolite profiling showed that CoCl_2_ treatment led to notable increases in glucose uptake, lactate, pyruvate, phosphoenolpyruvate (PEP), and glucose-6-phosphate (G6P), all of which were substantially reversed by Curcumol (Figure 4). These results indicate that Curcumol significantly impairs the glycolytic capacity of CRC cells and disrupts their metabolic adaptation under hypoxic conditions.

### 3.4. Curcumol Inhibits Energy Metabolism by Suppressing Glycolysis-Related Enzymes

**Figure 5:**
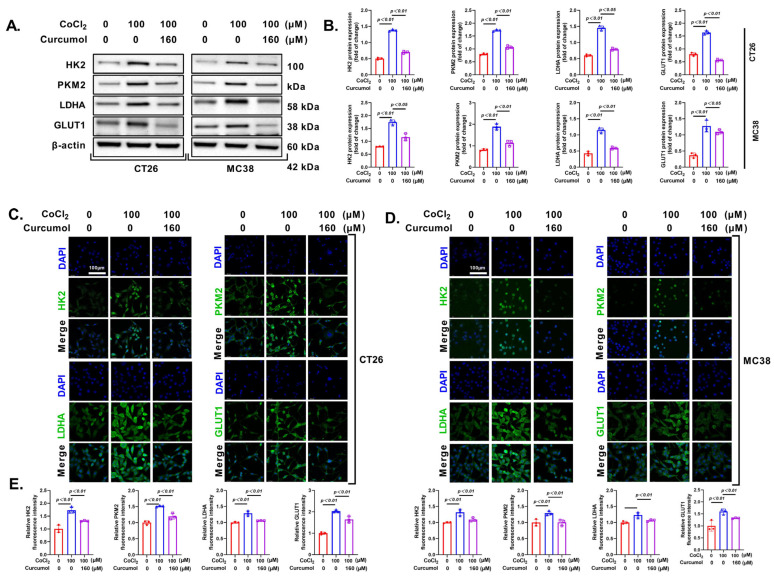
Curcumol inhibited energy metabolism by suppressing glycolysis-related enzymes. Cells were treated with Curcumol at indicated concentrations for 24 h. (**A**,**B**) Western blot analysis of HK2, PKM2, LDHA, GLUT1 with quantification. (**C**,**D**) IF analysis of HK2, PKM2, LDHA, GLUT1 with quantification. (**E**) Real-time PCR analysis of HK2, PKM2, LDHA, GLUT1. The results are presented as means ± SD. All experimental military repetitions three times (n = 3). Significance: CoCl_2_ vs. vector, CoCl_2_ vs. CoCl_2_ + Curcumol. The original images of the Western Blotting figures can be found in Supplementary Figure S2.

Next, we investigated the expression of key enzymes in the glycolytic pathway. The results of the Western blot analysis showed that Curcumol significantly inhibited the expression of crucial glycolytic enzymes, including HK2, PKM2, LDHA, and GLUT1 (Figure 5A,B). Consistent results were obtained from immunofluorescence (IF) and PCR analyses, further indicating that Curcumol blocks the glycolytic pathway in tumor cells to limit energy acquisition (Figure 5C–E). This finding aligns with the previously observed metabolic inhibition.

### 3.5. Curcumol Regulates Glycolytic Metabolism by Inhibiting HIF-1α

Given that key glycolytic enzymes such as HK2, PKM2, LDHA, and GLUT1 are well-established downstream targets transcriptionally activated by HIF-1α [25,26], and that previous studies have demonstrated that HIF-1α-mediated induction of glycolytic gene expression during hypoxic responses promotes metabolic reprogramming in tumor cells [27], we hypothesized that Curcumol’s inhibitory effect on glycolysis may be related to its regulation of the HIF-1α signaling axis. To determine whether Curcumol suppresses glycolysis by targeting hypoxia-associated transcription factors, we further investigated whether Curcumol downregulates the expression and activity of HIF-1α to exert its inhibitory effect on glycolytic metabolism. We examined its effects on HIF-1α expression in CT26 and MC38 CRC cells under CoCl_2_-induced hypoxic conditions.

**Figure 6:**
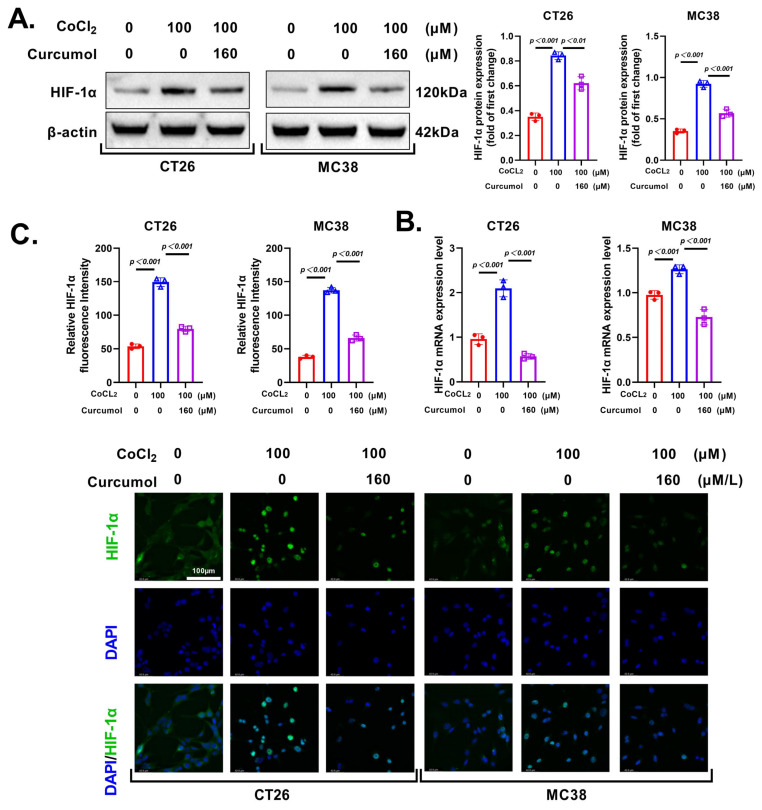
Curcumol regulated glycolytic metabolism by inhibiting HIF-1α. Cells were treated with Curcumol at indicated concentrations for 24 h. (**A**) Western blot analysis of HIF-1α with quantification. (**B**) Real-time PCR analysis of HIF-1α. (**C**) IF analysis of HIF-1α. The results are presented as means ± SD. All experimental military repetitions three times (n = 3). Significance: CoCl_2_ vs. vector, CoCl_2_ vs. CoCl_2_ + Curcumol. The original images of the Western Blotting figures can be found in Supplementary Figure S3.

Western blot analysis revealed that CoCl_2_ treatment significantly elevated HIF-1α protein levels, while co-treatment with Curcumol (160 μM) markedly suppressed this upregulation in both cell lines (Figure 6A). qRT-PCR analysis showed similar trends at the mRNA level, indicating that Curcumol also inhibits HIF-1α transcription (Figure 6B).

Immunofluorescence staining further confirmed that Curcumol significantly reduced nuclear HIF-1α expression under hypoxia-mimetic conditions (Figure 6C). Quantification of fluorescence intensity validated this result in both CT26 and MC38 cells.

In summary, Curcumol suppresses hypoxia-induced glycolytic reprogramming by downregulating HIF-1α expression, therefore impairing the metabolic adaptability of CRC cells.

### 3.6. HIF-1α Activation Rescues Curcumol-Induced Glycolytic Suppression

**Figure 7:**
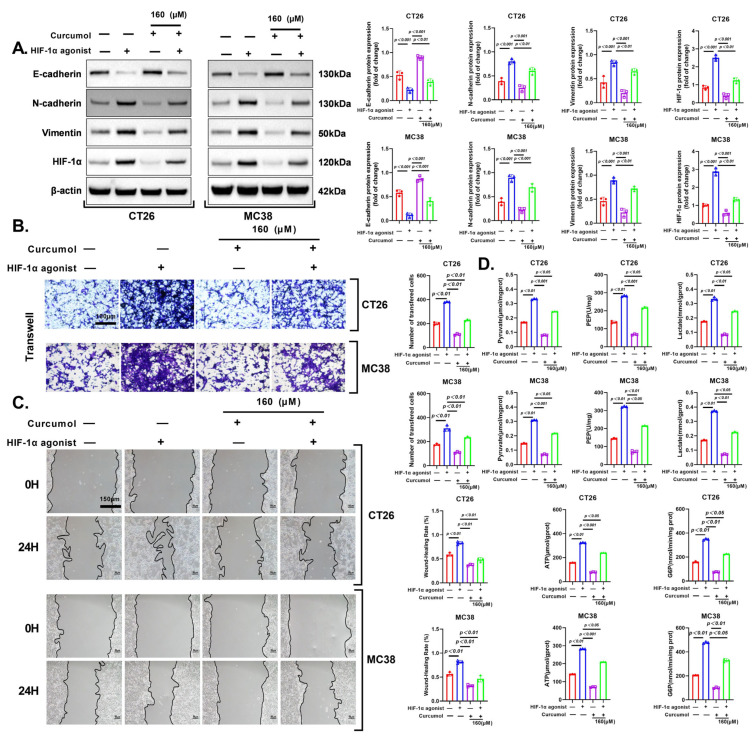
Curcumol regulated CRC progression via HIF-1α. Cells were treated with Curcumol at indicated concentrations for 24 h. (**A**) Western blot analysis of E-cadhernin, N-cadherinin, Vimentin, and HIF-1α with quantification. (**B**) Transwell invasion assay. (**C**) Wound healing assay with quantification. (**D**) Lactate, ATP, pyruvate, PEP, G6P analysis. The results are presented as means ± SD. All experimental military repetitions three times (n = 3). Significance: HIF-1α agonist (−) + Curcumol (−) vs. HIF-1α agonist (+) + Curcumol (−), HIF-1α agonist (−) + Curcumol (+) vs. HIF-1α agonist (+) + Curcumol (+), HIF-1α agonist (+) + Curcumol (−) vs. HIF-1α agonist (+) + Curcumol (+). (−) indicates the group without intervention of HIF-1α agonist or Curcumol; (+) indicates the group treated with HIF-1α agonist or Curcumol. The original images of the Western Blotting figures can be found in Supplementary Figure S4.

To determine whether the anti-tumor effects of Curcumol are mediated via inhibition of HIF-1α signaling, we used dimethyloxalylglycine (DMOG), a prolyl hydroxylase inhibitor that stabilizes HIF-1α. Western blot analysis showed that DMOG restored HIF-1α expression in both CT26 and MC38 cells following Curcumol treatment. In parallel, Curcumol-induced upregulation of E-cadherin and downregulation of N-cadherin and Vimentin were partially reversed by DMOG, indicating that EMT suppression by Curcumol is HIF-1α-dependent (Figure 7A).

Functionally, Transwell invasion assays demonstrated that DMOG significantly rescued the inhibitory effects of Curcumol on invasive capacity (Figure 7B). Consistently, wound healing assays revealed that DMOG treatment partially reversed Curcumol-induced migration inhibition under hypoxic-mimetic conditions (Figure 7C).

Furthermore, metabolic profiling showed that Curcumol significantly reduced levels of key glycolytic metabolites including lactate, ATP, pyruvate, phosphoenolpyruvate (PEP), and glucose-6-phosphate (G6P), while co-treatment with DMOG effectively restored these metabolic changes (Figure 7D).

In summary, these results indicate that HIF-1α plays a critical role in mediating the anti-EMT, anti-invasive, and anti-glycolytic effects of Curcumol, supporting the conclusion that Curcumol suppresses CRC progression through inhibition of the HIF-1α pathway.

### 3.7. Curcumol Enhances HIF-1α Degradation via VHL Pathway

To elucidate how Curcumol regulates HIF-1α degradation, we investigated its impact on the PHD–VHL pathway under hypoxia-mimetic conditions. HIF-1α stability is post-translationally regulated by hydroxylation via prolyl hydroxylase domain proteins (PHDs), which facilitates its recognition by the von Hippel–Lindau (VHL) E3 ubiquitin ligase complex, leading to proteasomal degradation under normoxia [28,29,30,31]. Disruption of this pathway results in HIF-1α accumulation and downstream glycolytic activation.

**Figure 8:**
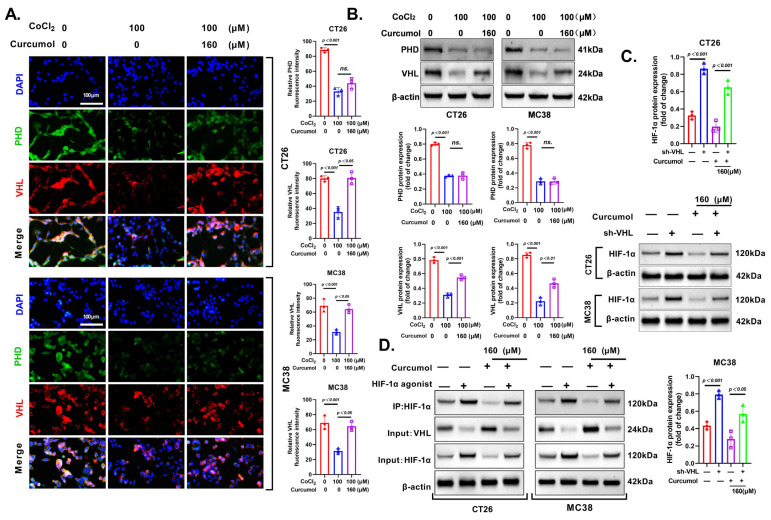
Curcumol enhanced HIF-1α degradation via the VHL pathway. Cells were treated with Curcumol at indicated concentrations for 24 h. (**A**) Immunofluorescence co-localization of PHD and VHL. (**B**) Western blot analysis of PHD and VHL protein expression in CT26 and MC38 cells. (**C**) Western blot analysis of VHL and HIF-1α. (**D**) Co-immunoprecipitation analysis of the interaction between HIF-1α and VHL proteins. The results are presented as means ± SD. All experimental military repetitions three times (n = 3). Significance: CoCl_2_ vs. vector, CoCl_2_ vs. CoCl_2_ + Curcumol, HIF-1α agonist (−) + Curcumol (−) vs. HIF-1α agonist (+) + Curcumol (−), HIF-1α agonist (−) + Curcumol (+) vs. HIF-1α agonist (+) + Curcumol (+). (−) indicates the group without intervention of HIF-1α agonist or Curcumol; (+) indicates the group treated with HIF-1α agonist or Curcumol The original images of the Western Blotting figures can be found in Supplementary Figure S5.

Immunofluorescence staining revealed that Curcumol significantly increased VHL expression in both CT26 and MC38 cells even under CoCl_2_-induced hypoxia, whereas its effect on PHD levels was minimal and not statistically significant (Figure 8A). These findings suggest that Curcumol promotes HIF-1α degradation primarily by modulating VHL expression rather than upstream PHD activity.

Western blot analysis further confirmed that VHL protein levels were significantly upregulated by Curcumol but remained unaffected in terms of PHD expression (Figure 8B). Importantly, knockdown of VHL abolished the Curcumol-induced suppression of HIF-1α expression in both CT26 and MC38 cells (Figure 8C), indicating that the inhibitory effect of Curcumol on HIF-1α is largely dependent on the presence of VHL.

Co-immunoprecipitation assays were performed to evaluate whether Curcumol promotes the interaction between VHL and HIF-1α. The results showed enhanced VHL–HIF-1α binding following Curcumol treatment, which was diminished when cells were co-treated with HIF-1α agonist (Figure 8D).

In summary, these findings indicate that Curcumol facilitates HIF-1α degradation predominantly by upregulating VHL and enhancing its interaction with HIF-1α, therefore disrupting glycolytic signaling and limiting CRC progression.

### 3.8. Curcumol Reverses CoCl_2_-Induced Glycolytic Activation and EMT by Restoring the VHL/HIF-1α Axis

**Figure 9:**
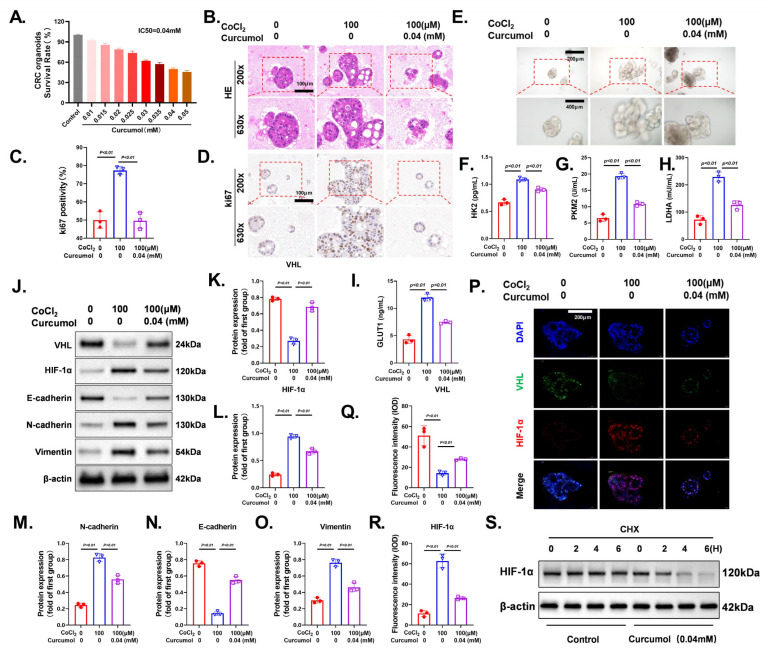
Curcumol reverses CoCl_2_-induced glycolytic activation and EMT via the VHL/HIF-1α axis. CRC organoids were treated with 100 μM CoCl_2_ and/or Curcumol (0.04 mM) for 24 h. (**A**) Organoid viability curve showing the IC_50_ of Curcumol under hypoxia-mimicking conditions. (**B**) H&E staining images of organoids from different treatment groups. (**C**,**D**) Ki-67 immunohistochemistry and quantification. (**E**) Brightfield images illustrating CoCl_2_-induced structural integrity of organoids and Curcumol-mediated disruption. (**F**–**I**) ELISA and quantitative analysis of key glycolytic enzymes: HK2, PKM2, LDHA, and GLUT1. (**J**) Western blot analysis of VHL, HIF-1α, E-cadherin, N-cadherin, and Vimentin expression. (**K**–**O**) Corresponding densitometric quantifications. (**P**) Immunofluorescence staining of VHL and HIF-1α with DAPI nuclear counterstaining. (**Q**,**R**) Quantitative analysis of VHL and HIF-1α fluorescence intensity. (**S**) Cycloheximide (CHX) experiment. All data are presented as means ± standard deviation (SD). All experimental military repetitions three times (n = 3). Statistical comparisons were made between the CoCl_2_ group and control, and between the CoCl_2_ group and the CoCl_2_ + Curcumol group. The original images of the Western Blotting figures can be found in Supplementary Figure S6.

To investigate the effect of Curcumol on hypoxia-induced malignant phenotypes, we first determined the half-maximal inhibitory concentration (IC_50_) of Curcumol in CRC organoids. Organoid viability assays revealed that Curcumol decreased organoid survival in a dose-dependent manner, with an IC_50_ of approximately 0.04 mM (Figure 9A). Histological and immunohistochemical analyses showed that treatment with CoCl_2_ (100 μM) significantly increased organoid volume and promoted cell proliferation, as indicated by preserved organoid structure on H&E staining (Figure 9B) and increased Ki-67 positivity (Figure 9D). Co-treatment with Curcumol (0.04 mM) significantly inhibited these proliferative effects, and quantitative analysis of Ki-67 confirmed the suppressive effect of Curcumol on CoCl_2_-induced proliferation (Figure 9C).

Morphologically, CoCl_2_-treated organoids exhibited increased volume and structural integrity, while Curcumol treatment significantly disrupted their structural integrity (Figure 9E). ELISA-based metabolic profiling demonstrated that CoCl_2_ significantly upregulated key glycolytic enzymes, including HK2, PKM2, LDHA, and GLUT1. In contrast, Curcumol effectively suppressed the expression of these metabolic enzymes, suggesting its ability to inhibit hypoxia-induced glycolytic reprogramming (Figure 9F–I).

Mechanistically, Western blot analysis revealed that CoCl_2_ markedly downregulated VHL expression while upregulating HIF-1α, N-cadherin, and Vimentin, alongside suppression of E-cadherin, indicating activation of the EMT process. Curcumol treatment reversed these molecular alterations, significantly restoring VHL expression, suppressing HIF-1α accumulation, and correcting EMT-related protein expression (Figure 9J). Densitometric analysis further validated the significant regulatory effect of Curcumol on these proteins (Figure 9K–O).

Immunofluorescence staining provided additional validation. In the CoCl_2_ group, VHL expression was notably reduced, while HIF-1α accumulated in the nucleus. Curcumol treatment significantly enhanced the VHL signal and reduced HIF-1α nuclear localization (Figure 9P). Quantitative results of fluorescence intensity confirmed that Curcumol effectively reversed VHL suppression and HIF-1α activation (Figure 9Q,R). To further explore the mechanism underlying HIF-1α reduction, we conducted a cycloheximide (CHX) chase assay to evaluate its protein stability. HIF-1α protein levels remained relatively stable in control cells, whereas Curcumol treatment markedly accelerated HIF-1α degradation over time (Figure 9S). These findings indicate that Curcumol facilitates HIF-1α destabilization, possibly through the VHL-mediated ubiquitin–proteasome pathway.

In summary, these findings indicate that Curcumol significantly inhibits CoCl_2_-induced glycolytic enhancement and EMT by restoring VHL expression and suppressing HIF-1α signaling, thereby reversing the hypoxia-driven malignant phenotype in CRC organoids.

### 3.9. Curcumol Exhibits Potent and Dose-Dependent Anti-Tumor Effects In Vivo

**Figure 10:**
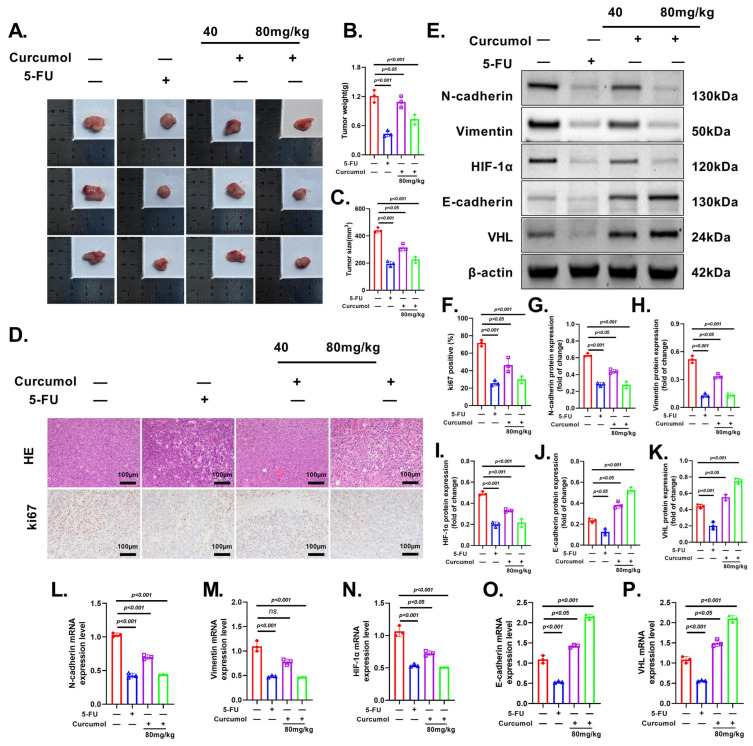
Curcumol exhibited potent and dose-dependent anti-tumor effects in vivo. (**A**) Representative images of tumors from orthotopic CRC mouse models treated with vehicle, low-dose (40 mg/kg), and high-dose (80 mg/kg) Curcumol. (**B**) Tumor volume and (**C**) tumor weight were measured after 14 days of treatment. (**D**,**F**) H&E staining and Ki-67 immunohistochemistry of tumor tissues showing necrotic regions and cell proliferation status. (**E**,**G**–**K**) Western blot analysis. (**L**–**P**) qRT-PCR analysis of metastasis-related proteins and key regulators of the VHL/HIF-1α pathway. The results are presented as means ± SD. All experimental military repetitions three times (n = 3). Significance: control vs. Curcumol. (−) indicates the group without intervention of 5-FU or Curcumol; (+) indicates the group treated with 5-FU or Curcumol. The original images of the Western Blotting figures can be found in Supplementary Figure S7.

To investigate the in vivo anti-tumor effects of Curcumol and its dose dependency, an orthotopic CRC mouse model was established and treated with low-dose (40 mg/kg) or high-dose (80 mg/kg) Curcumol. Gross tumor morphology, volume, and weight measurements demonstrated that Curcumol significantly inhibited tumor growth, with more pronounced effects observed in the high-dose group, indicating a clear dose-dependent trend (Figure 10A–C). Histological analysis of H&E staining showed increased necrotic regions and reduced cellular density in tumor tissues following Curcumol treatment. Ki-67 immunohistochemistry further confirmed that Curcumol exerted a strong anti-proliferative effect, which was more evident at the higher dose (Figure 10D,F). Western blot and qRT-PCR analyses revealed that Curcumol downregulated the expression of metastasis-related proteins in a dose-dependent manner, significantly suppressed HIF-1α expression, and changed VHL levels accordingly (Figure 10E,G–P). These findings suggest that the anti-tumor effects of Curcumol in vivo might be closely associated with its ability to regulate the VHL/HIF-1α signaling axis and inhibit the invasive potential of CRC cells.

### 3.10. Curcumol Exerts Anti-Tumor Effects via VHL/HIF-1α Pathway

**Figure 11:**
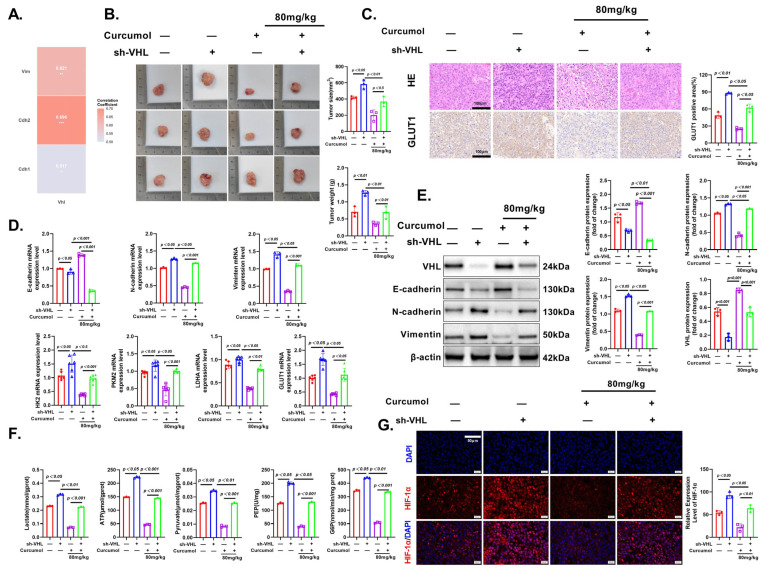
VHL is required for Curcumol suppression of CRC growth and metastasis in mice. Nude mice were transfected with VHL knockout plasmid and induced CRC model. See the Methods section for details. (**A**) Transcriptome-based Pearson correlation analysis shows that VHL expression is significantly correlated with glycolysis- and EMT-related genes, including Vim, Cdh2, and Cdh1 (*p* < 0.05). (**B**) Measurements of tumor morphology and weight. (**C**) HE and IHC analysis of GLUT1 with quantification. (**D**) Real-time PCR analysis of HK2, PKM2, LDHA, GLUT1 with quantification. (**E**) Western blot analysis of VHL, E-cadherin, N-cadherin, and Vimentin. (**F**) Lactate, ATP, pyruvate, PEP, G6P analysis. (**G**) IF of HIF-1α with quantification. The results are presented as means ± SD. All experimental military repetitions three times (n = 3). Significance: sh-VHL vs. vector, sh-VHL vs. sh-VHL + Curcumol. (−) indicates the group without intervention of sh-VHL or Curcumol; (+) indicates the group treated with sh-VHL or Curcumol. * 0.517, ** 0.621, *** 0.696. The original images of the Western Blotting figures can be found in Supplementary Figure S8.

Finally, we validated the anti-tumor effects of Curcumol in an orthotopic CRC model and investigated the role of VHL. Tumor morphology, volume, and weight measurements confirmed that Curcumol effectively inhibited tumor growth, while VHL knockdown promoted tumor progression and reversed the inhibitory effects of Curcumol (Figure 11B). H&E staining revealed more extensive necrotic areas in the Curcumol-treated group, while VHL deficiency reversed these histological changes, consistent with the tumor growth data (Figure 11C). IHC results showed that Curcumol treatment significantly downregulated the expression of GLUT1 in tumor tissues, suggesting its regulatory effect on glucose metabolism. This inhibitory effect was significantly attenuated upon VHL knockdown (Figure 11C).

To further elucidate the molecular correlations underlying these effects, we performed transcriptome-based correlation analysis. Pearson correlation analysis further demonstrated that the expression levels of Vim, Cdh2, and Cdh1 were significantly correlated with the expression of VHL (*p* < 0.05) (Figure 11A). Meanwhile, IF staining demonstrated that Curcumol suppressed both the expression and nuclear accumulation of HIF-1α, indicating interference with hypoxia-related signaling. However, this effect was partially reversed in the absence of VHL (Figure 11G). RT-PCR analysis demonstrated that Curcumol downregulated the mRNA expression of glycolysis-related genes (HK2, PKM2, LDHA, GLUT1) and EMT markers (E-cadherin, N-cadherin, Vimentin), and these effects were attenuated in the absence of VHL (Figure 11D). Western blot analysis confirmed these findings at the protein level (Figure 11E). Furthermore, metabolic profiling showed that Curcumol reduced the levels of glycolytic metabolites, including lactate, ATP, pyruvate, PEP, and G6P, and VHL knockdown reversed these effects (Figure 11F). These results confirm that Curcumol exerts strong anti-tumor effects by modulating the VHL/HIF-1α axis and disrupting glycolysis and EMT progression in CRC.

## 4. Discussion

This study is the first to reveal that Curcumol suppresses the progression of colorectal cancer (CRC) by activating the VHL/HIF-1α signaling pathway, thereby significantly inhibiting hypoxia-driven glycolysis. Under hypoxic conditions, HIF-1α becomes stabilized and induces the upregulation of multiple key glycolytic enzymes, promoting metabolic reprogramming and enhancing the invasive potential of CRC cells [32,33,34]. In this study, we established hypoxia models in CT26 and MC38 cells using CoCl_2_ treatment and found that Curcumol markedly downregulated HIF-1α expression at both the transcriptional and protein levels. This was accompanied by decreased expression of key glycolytic enzymes such as HK2, LDHA, and GLUT1, along with reduced levels of glycolysis-related metabolites including lactate and ATP, indicating a strong inhibitory effect on the glycolytic pathway.

Metabolic plasticity in tumor cells is a key mechanism through which they adapt to hypoxic stress and develop resistance to therapy [35]. This metabolic reprogramming not only supplies energy for tumor growth but also leads to acidification of the tumor microenvironment [36,37] and contributes to immune suppression [38,39,40,41]. The Warburg effect, characterized by a preference for glycolysis even under normoxic conditions, is a prominent phenotype driven by HIF-1α. The stability of HIF-1α is critically regulated by the tumor suppressor VHL [42,43,44]. Our study further confirmed that this effect is dependent on VHL expression. Curcumol not only significantly upregulated VHL protein levels but also enhanced the interaction between VHL and HIF-1α, promoting HIF-1α degradation. Notably, in the VHL-knockout CT26 model, the inhibitory effects of Curcumol on HIF-1α, glycolytic metabolism, and EMT were almost completely abolished, further validating the VHL-dependent mechanism of its action.

In addition to suppressing tumor metabolism, we found that Curcumol also markedly reversed hypoxia-induced epithelial–mesenchymal transition (EMT), a key step in the metastatic cascade of colorectal cancer (CRC) [45]. Upon CoCl_2_ treatment, both CRC cells and organoids exhibited classic EMT features, characterized by reduced E-cadherin expression and elevated N-cadherin and Vimentin levels. Curcumol treatment effectively reversed these changes at both the transcriptional and protein levels. Furthermore, scratch wound and Transwell assays demonstrated that Curcumol significantly inhibited hypoxia-induced cell migration and invasion. Importantly, administration of the HIF-1α agonist DMOG partially reversed the inhibitory effects of Curcumol on both EMT and glycolysis, further underscoring the central role of the HIF-1α signaling pathway in mediating these processes.

In the organoid model, we observed that under CoCl_2_-induced hypoxic conditions, Curcumol inhibited the viability of CRC organoids in a dose-dependent manner, disrupted their structural integrity, and modulated the expression of EMT markers. These findings were further validated in an in vivo orthotopic implantation model. Curcumol exhibited a clear dose-dependent anti-tumor effect in vivo, as evidenced by reduced tumor burden and decreased Ki-67 expression. However, under VHL knockout conditions, the anti-tumor efficacy of Curcumol was markedly attenuated, as reflected by increased tumor burden, enhanced glycolytic activity, and re-emergence of EMT phenotypes.

The translational significance of this study lies in the identification of Curcumol—a natural product derived from traditional Chinese medicine—as a promising metabolic intervention agent with favorable low-toxicity pharmacological properties. We provide systematic experimental evidence supporting its potential application in colorectal cancer (CRC) therapy. Importantly, the VHL/HIF-1α signaling axis emerges as a critical regulatory target, which not only mediates glycolytic suppression but may also serve as a therapeutic strategy to curb tumor metastasis.

Nevertheless, certain limitations should be acknowledged. The upstream molecular mechanism by which Curcumol regulates VHL expression remains unclear. Although its biological effects were validated in cellular, organoid, and animal models, further studies incorporating clinical samples and genetic models are necessary to confirm the mechanistic insights and clinical relevance.

**Figure 12:**
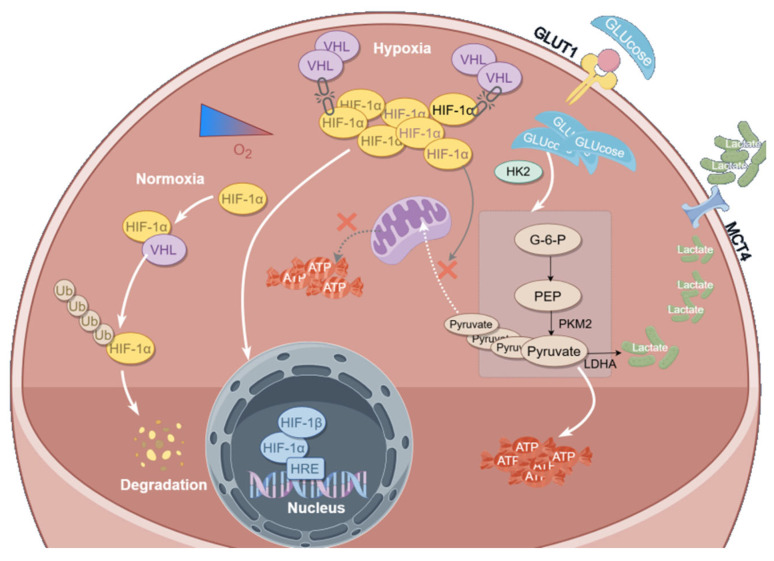
Mechanism.

In conclusion, this study systematically reveals for the first time that Curcumol inhibits glycolysis and colorectal cancer (CRC) progression by activating the VHL/HIF-1α signaling axis. These findings provide a novel mechanistic insight into targeting tumor metabolic reprogramming and underscore the therapeutic potential of natural compounds in CRC treatment (Figure 12).

## 5. Conclusions

In summary, our study demonstrates that Curcumol inhibits colorectal cancer (CRC) progression by activating the VHL/HIF-1α pathway, suppressing glycolysis and lactate production, and improving tumor microenvironment. Beyond metabolic regulation, Curcumol also modulates key oncogenic signaling pathways to limit tumor growth and survival. These findings highlight Curcumol’s potential as a novel therapeutic agent for CRC, supporting its further exploration in immune modulation and combinational strategies for personalized therapy.

## Figures and Tables

**Table 1:** VHL-CRISPR primer sequences.

Primer Name	Forward (5′-3′)	Reverse (5′-3′)
VHL-CRISPR	CACC GCGTTCCAATAATGCCCCGGA	AAAC TCCGGGGCATTATTGGAACGC

**Table 2:** Primer sequences.

Gene	Forward	Reverse
*β-actin*	ACTCTTCCAGCCTTCCTTCC	CGTCATACTCCTGCTTGCTG
*HIF-1α*	CTGCCACTGCCACCACAACTG	TGCCACTGTATGCTGATGCCTTAG
*HK2*	GACGAGAGCATCCTCCTCAAGTG	TCACCACAGCAACCACATCCAG
*PKM2*	TTGCCTGCTGTGTCGGAGAAG	CAGATGCCTTGCGGATGAATGAC
*LDHA*	TCAGCCCGATTCCGTTACCTAATG	CACCAGCAACATTCATTCCACTCC
*GLUT1*	GATGAAAGAAGAGGGTCGGCAGATG	CAGCACCACAGCGATGAGGATG

## Data Availability

The research data for this paper were obtained from a public database, as indicated in the text. The original data presented in the study are included in the article and are available from the corresponding authors upon reasonable request.

## Supplementary Materials

The following supporting information can be downloaded at: https://www.mdpi.com/article/10.3390/cancers17183000/s1: Figure S1: Original Western Blotting figures of Figure 2B; Figure S2: Original Western Blotting figures of Figure 5A; Figure S3: Original Western Blotting figures of Figure 6A; Figure S4: Original Western Blotting figures of Figure 7A; Figure S5: Original Western Blotting figures of Figure 8B; Figure S6: Original Western Blotting figures of Figure 9J; Figure S7: Original Western Blotting figures of Figure 10E; Figure S8: Original Western Blotting figures of Figure 11E.

## Abbreviations

The following abbreviations are used in this manuscript:

ATP	adenosine triphosphate
Co-IP	co-immunoprecipitation
CRC	colorectal cancer
ECAR	extracellular acidification rate
EMT	epithelial–mesenchymal transition
G6P	glucose-6-phosphate
GLUT1	glucose transporter 1
HIF-1α	hypoxia-inducible factor 1 alpha
HK2	hexokinase 2
IHC	immunohistochemistry
IF	immunofluorescence
LDHA	lactate dehydrogenase A
MAPK	mitogen-activated protein kinase
OCR	oxygen consumption rate
PEP	phosphoenolpyruvate
qRT-PCR	quantitative real-time polymerase chain reaction
VHL	von Hippel–Lindau protein
WB	Western blot
