# Supplementary figures and images for: Curcumol Targets the VHL/HIF-1α Axis to Suppress Glycolysis-Driven Progression in Colorectal Cancer
Source: Cancers (Basel). 2025 Sep 14;17(18):3000. doi: 10.3390/cancers17183000 (PMC12468461; doi:10.3390/cancers17183000)

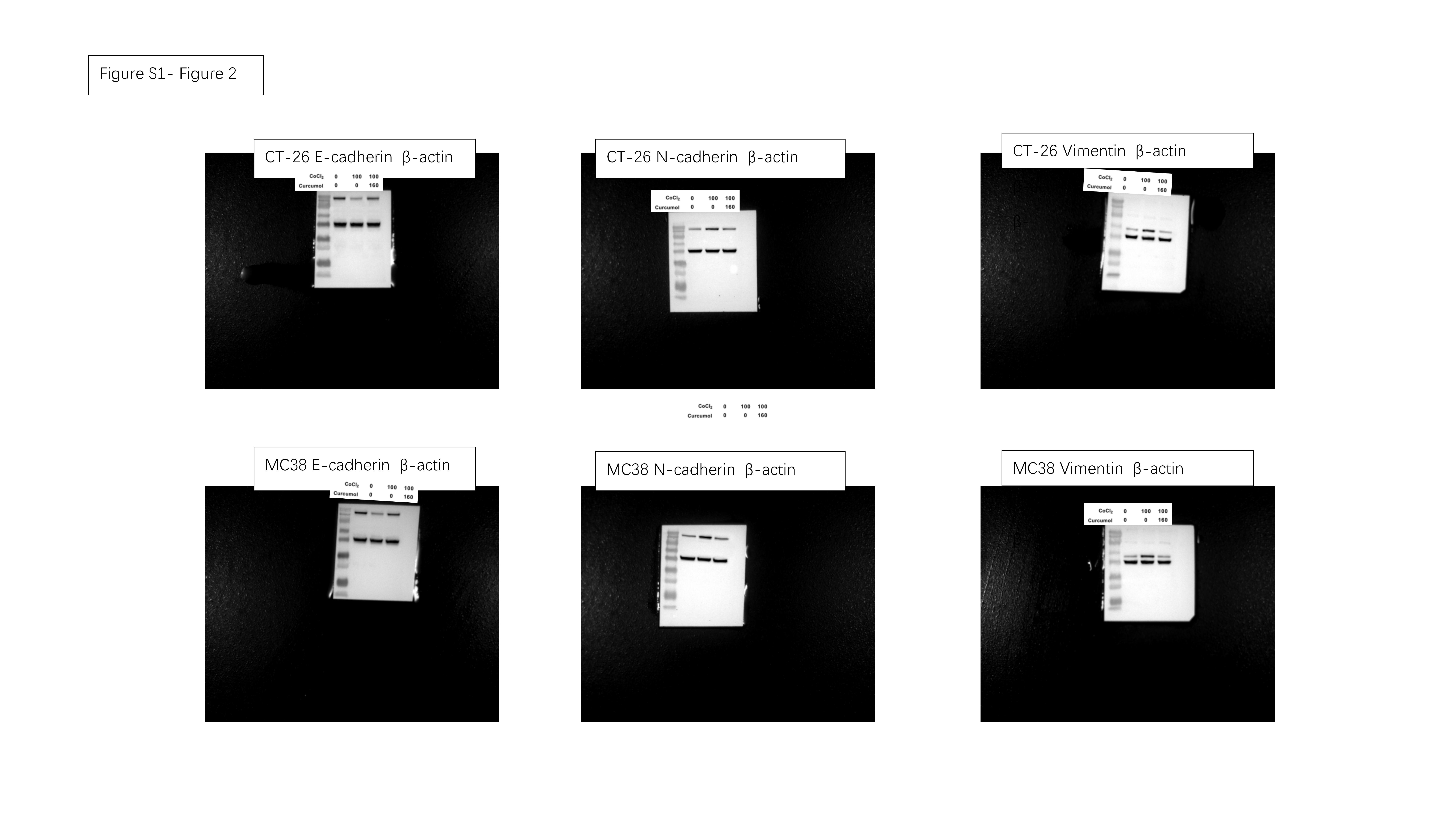

Supplement: Supplementary file 1 [file cancers-17-03000-s001.zip › Original images of the Western Blotting figures/Figure S1.jpg]

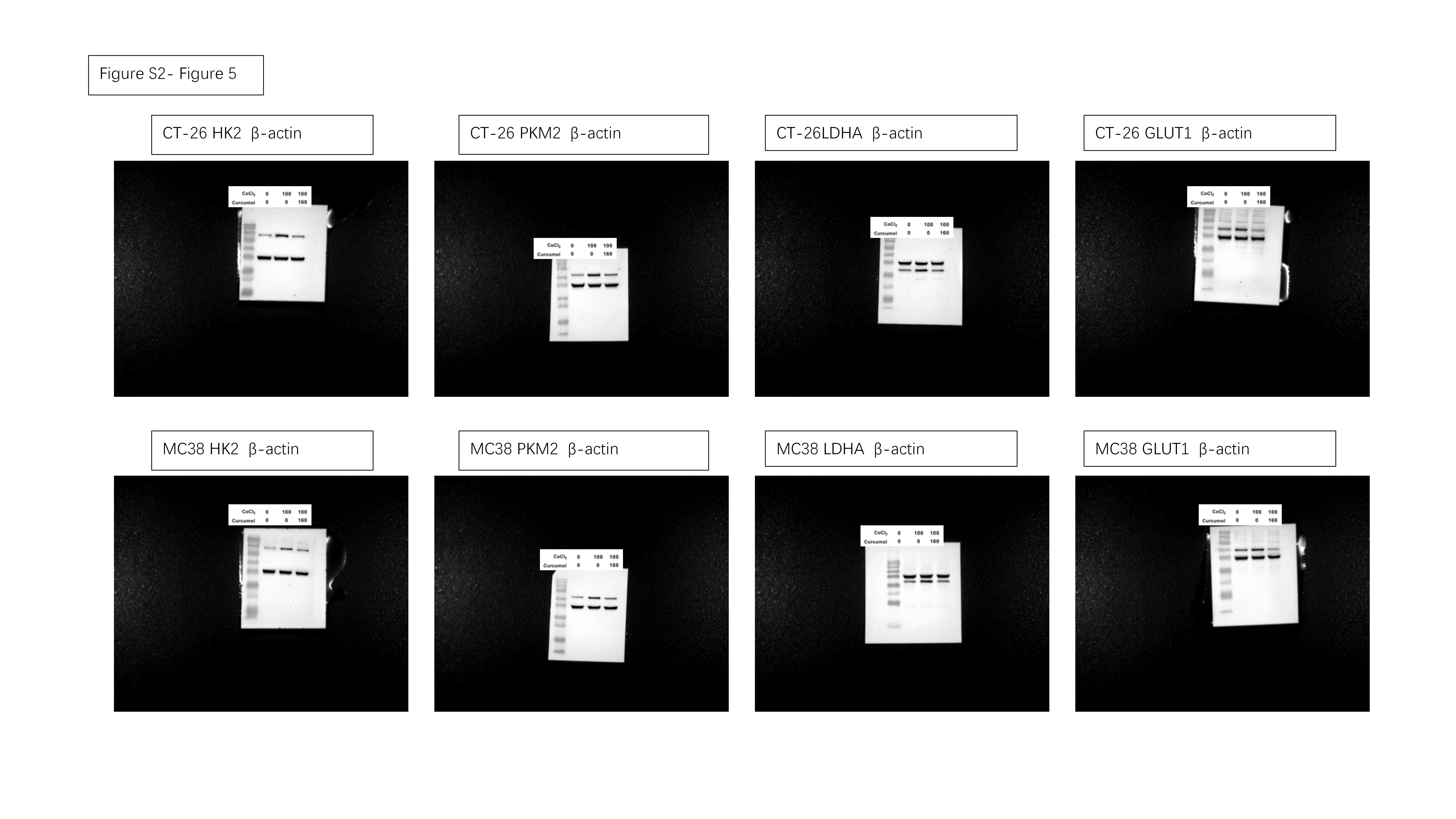

Supplement: Supplementary file 1 [file cancers-17-03000-s001.zip › Original images of the Western Blotting figures/Figure S2.jpg]

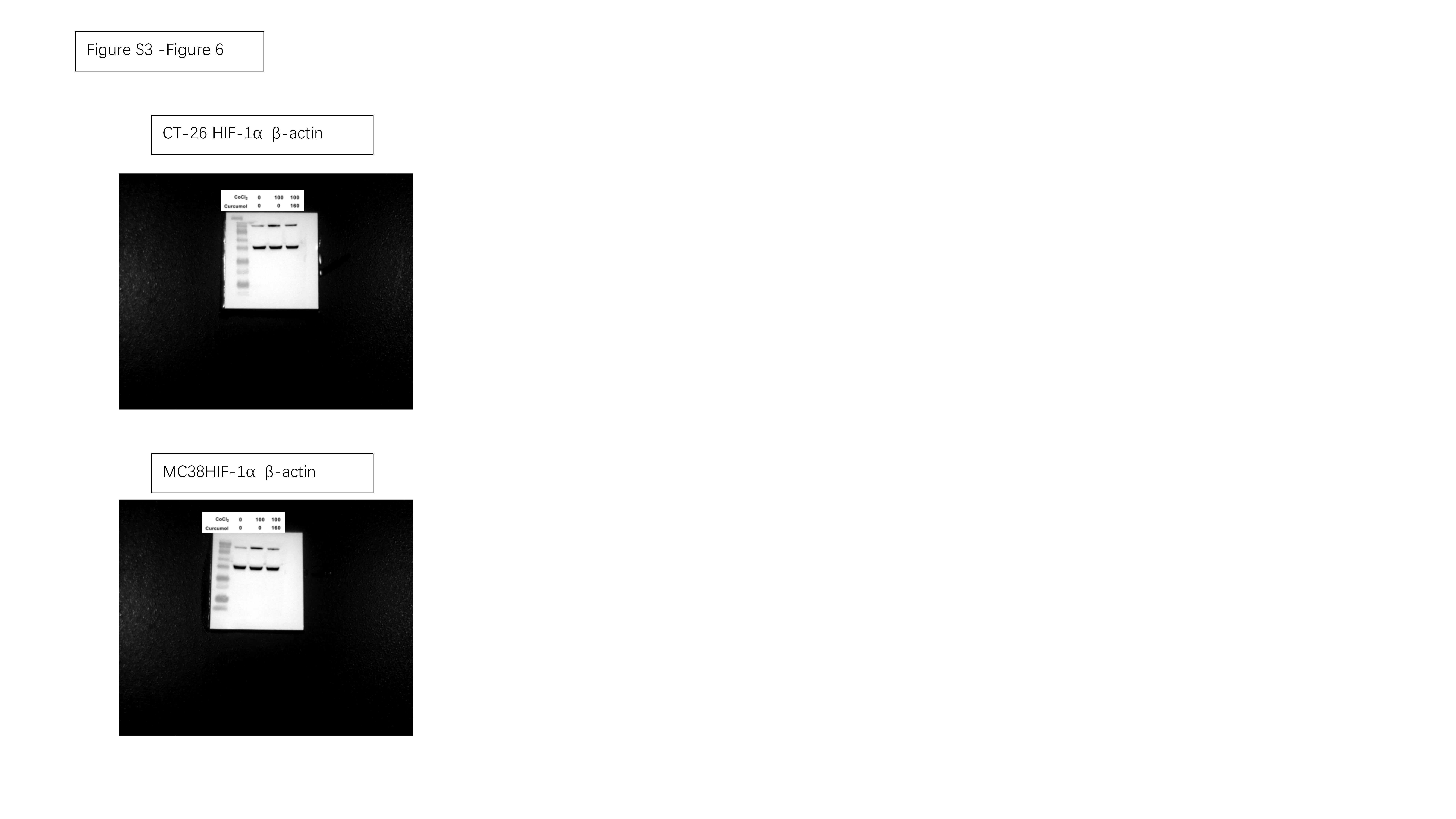

Supplement: Supplementary file 1 [file cancers-17-03000-s001.zip › Original images of the Western Blotting figures/Figure S3.jpg]

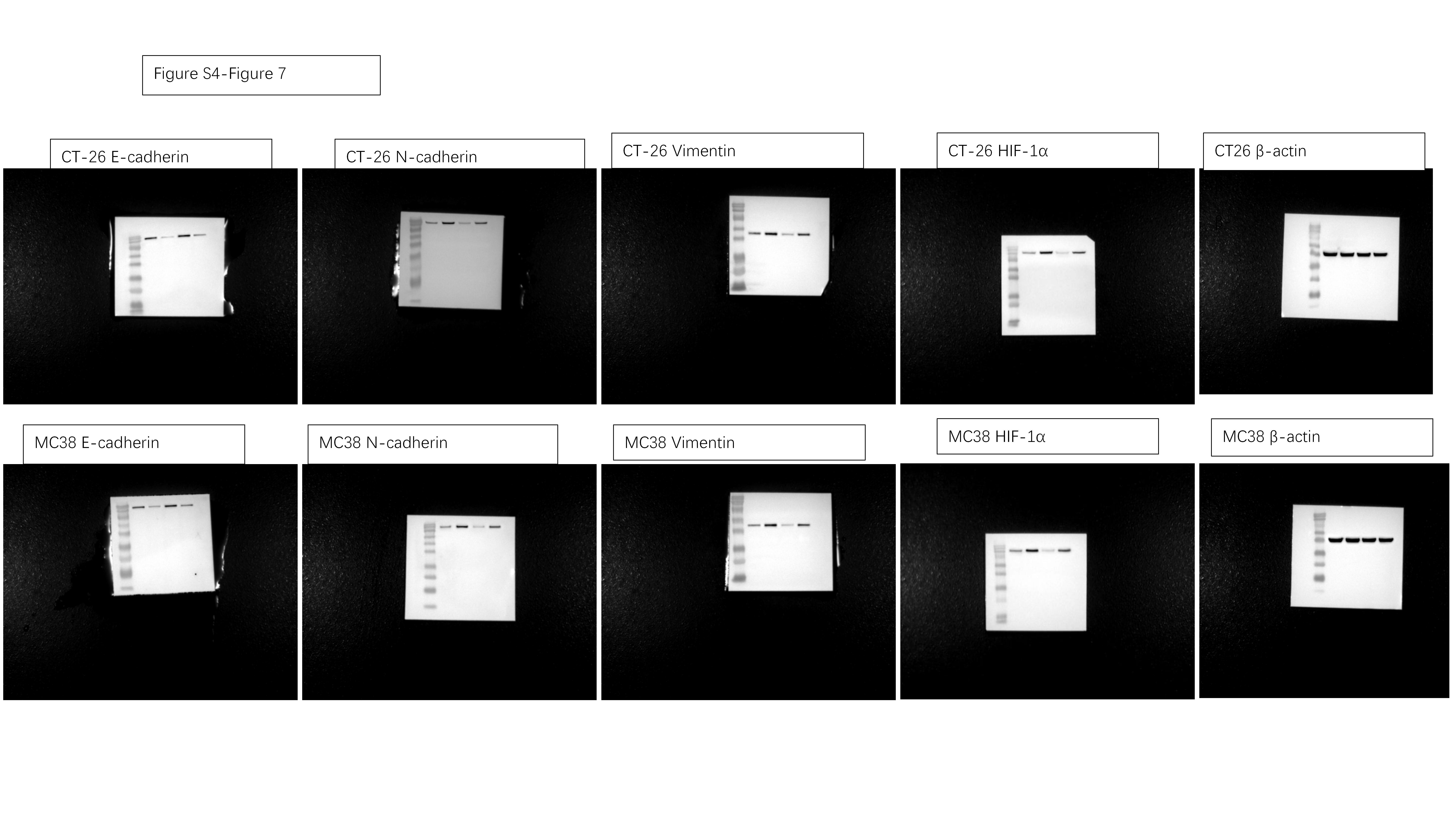

Supplement: Supplementary file 1 [file cancers-17-03000-s001.zip › Original images of the Western Blotting figures/Figure S4.jpg]

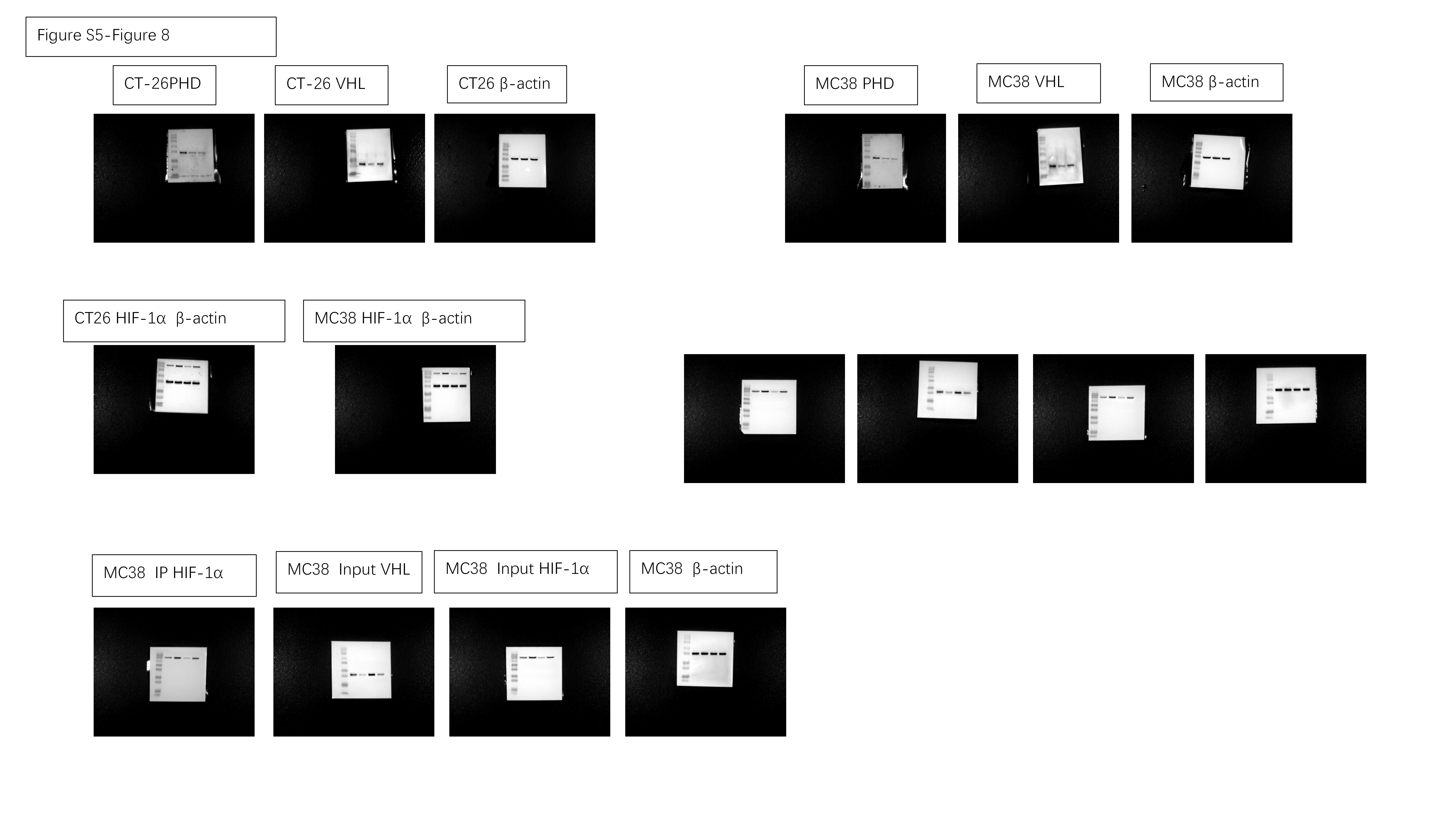

Supplement: Supplementary file 1 [file cancers-17-03000-s001.zip › Original images of the Western Blotting figures/Figure S5.jpg]

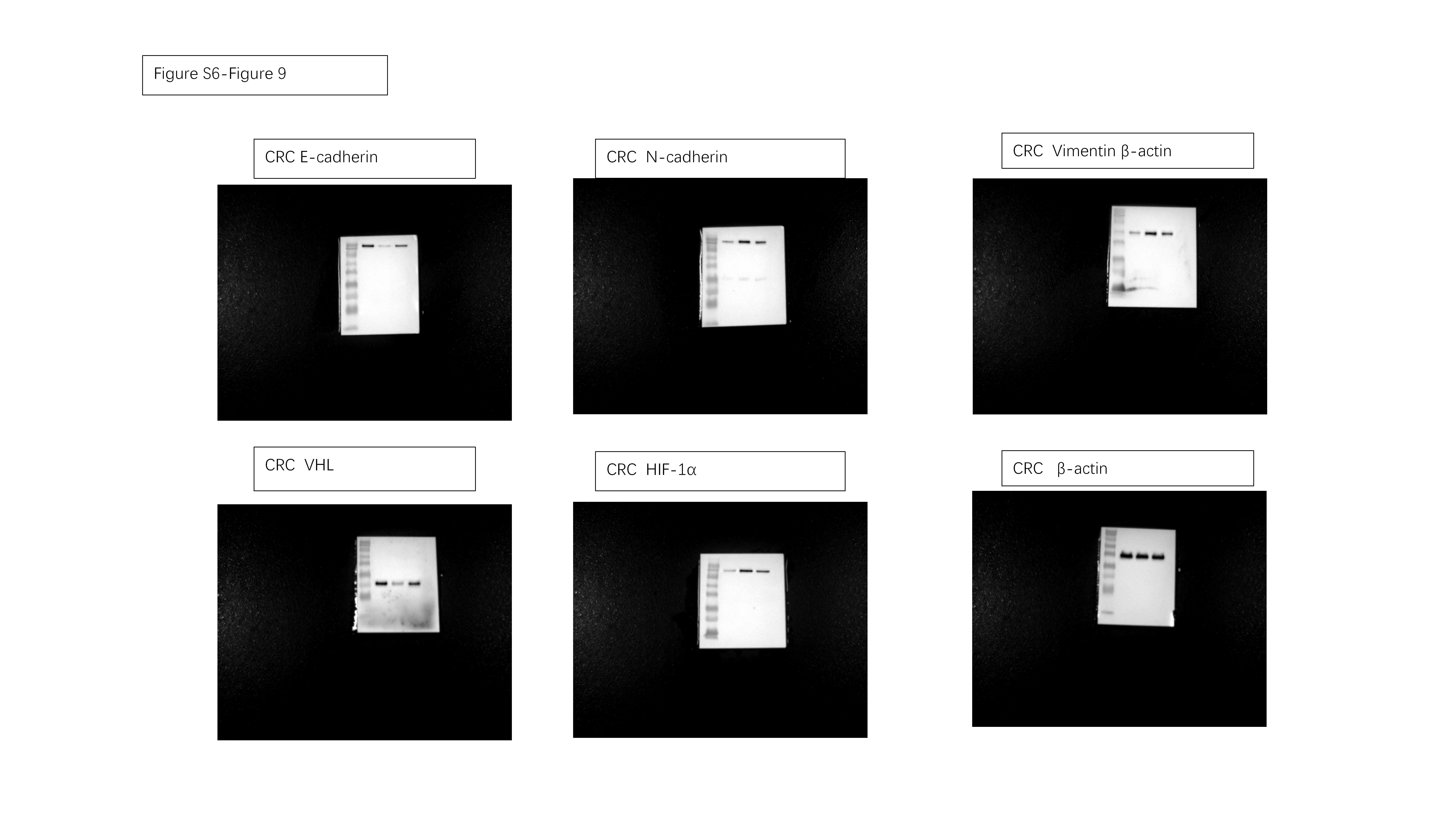

Supplement: Supplementary file 1 [file cancers-17-03000-s001.zip › Original images of the Western Blotting figures/Figure S6.jpg]

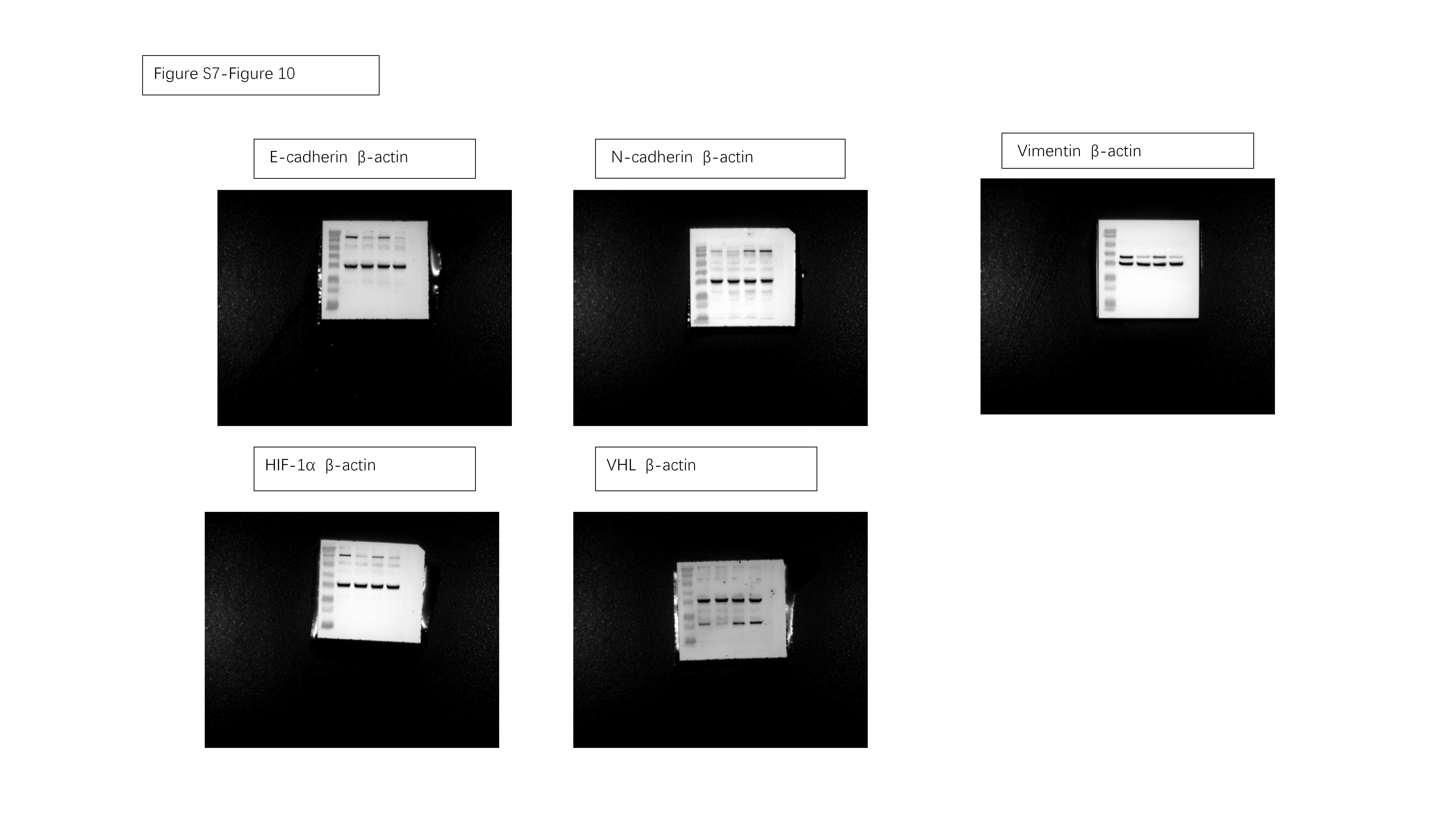

Supplement: Supplementary file 1 [file cancers-17-03000-s001.zip › Original images of the Western Blotting figures/Figure S7.jpg]

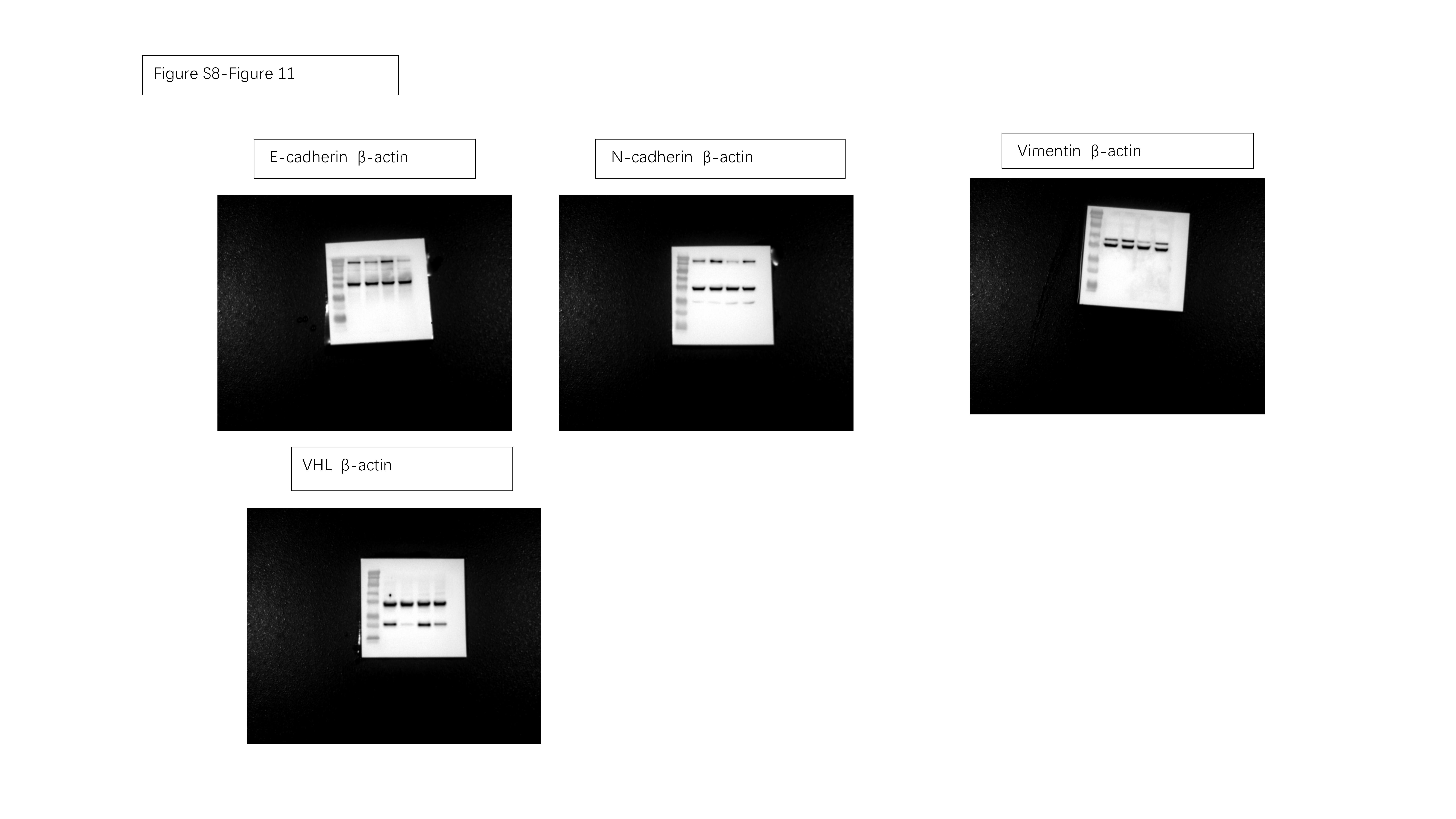

Supplement: Supplementary file 1 [file cancers-17-03000-s001.zip › Original images of the Western Blotting figures/Figure S8.jpg]
